# Correction: Mapping and role of T cell response in SARS-CoV-2–infected mice

**DOI:** 10.1084/jem.2020218710052021c

**Published:** 2021-10-15

**Authors:** Zhen Zhuang, Xiaomin Lai, Jing Sun, Zhao Chen, Zhaoyong Zhang, Jun Dai, Donglan Liu, Yuming Li, Fang Li, Yanqun Wang, Airu Zhu, Junxiang Wang, Wenhui Yang, Jicheng Huang, Xiaobo Li, Lingfei Hu, Liyan Wen, Jianfen Zhuo, Yanjun Zhang, Dingbin Chen, Suxiang Li, Shuxiang Huang, Yongxia Shi, Kui Zheng, Nanshan Zhong, Jingxian Zhao, Dongsheng Zhou, Jincun Zhao

Vol. 218, No. 4 | 10.1084/jem.20202187 | January 19, 2021

The authors regret that the amino acid sequence of one of the T epitopes in Table 1 was incorrect. The corrected table is shown here, with new data in red.

**Table 1.**
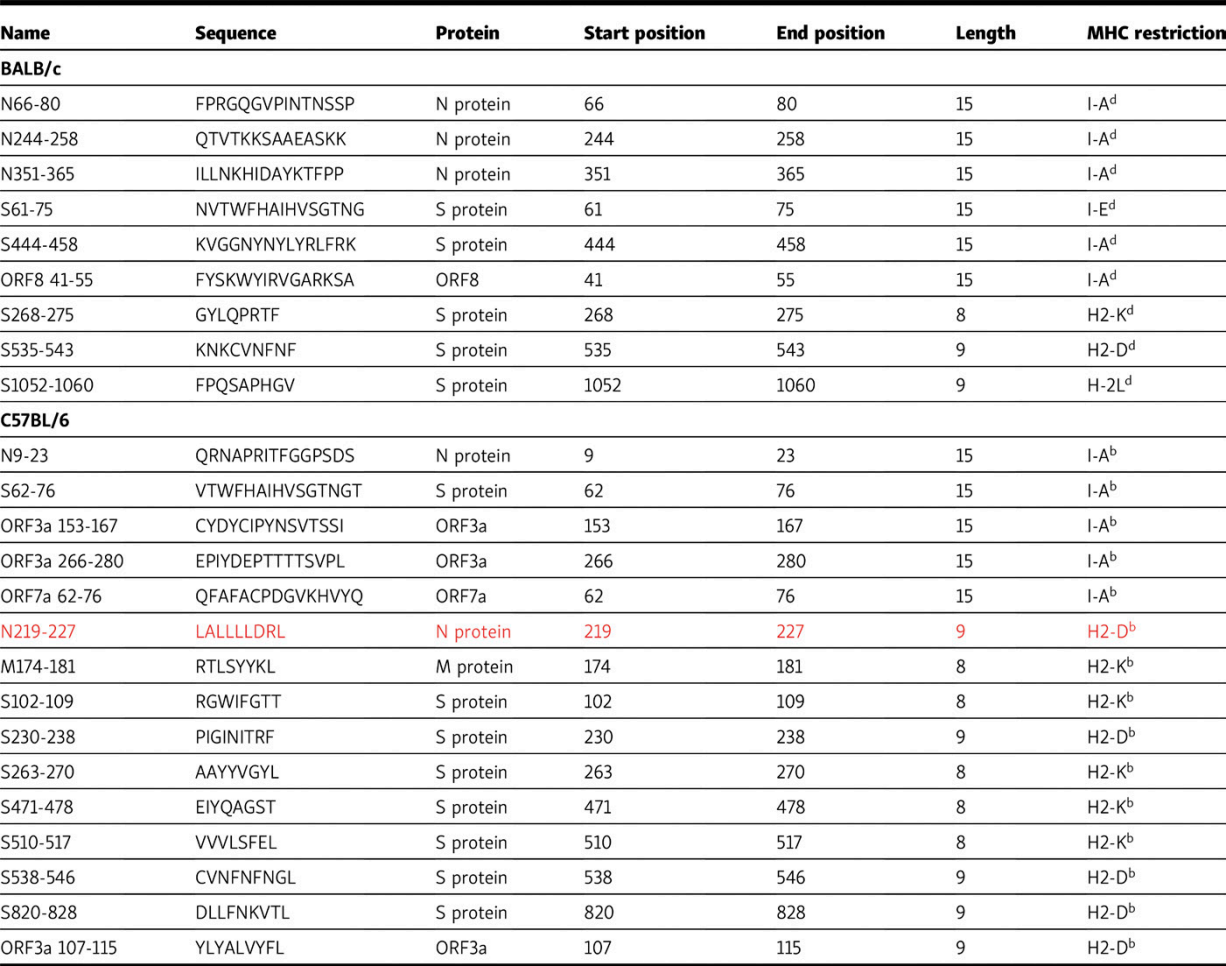
Characteristics of SARS-CoV-2–specific T cell epitopes in BALB/c mice and C57BL/6 mice

The error appears in print and in PDFs downloaded before September 30, 2021.

